# Hand hygiene improvement program in the Canary Islands, Spain

**DOI:** 10.1186/1753-6561-5-S6-P126

**Published:** 2011-06-29

**Authors:** J Molina, P Garcia

**Affiliations:** 1 Servicio Canario de la Salud, Las Palmas, Spain; 2 Servicio Canario de la Salud, Tenerife, Spain

## Introduction / objectives

To describe the evaluation of a coordinate campaign on hand hygiene (HH) improvement in the Healthcare System in the Canary Islands after the first year of implementation.

## Methods

### Setting

Ten public healthcare hospitals. A core team with a plan for implementation was set at the beginning.

### Intervention

Implementation of the WHO multimodal strategy for improving HH.

### Measures

Direct observation of hand hygiene for 1 and 2 moments (before patient contact and before aseptic task) in emergency and intensive care units and consumption of alcohol–based products for hand rub (liters / 1000 patient-days) every six months in two six-month’s period. Training was performed in each facility.

## Results

Alcohol-based hand rub products were made available at each centre. 1730 dispensers accounted for the 3861 available beds (44,8%). Seven out of ten hospitals implemented training about HH, but just four centers trained about the concept of “My Five Moments for Hand Hygiene”. Medical doctors had the lowest level of attendance.

Eight out of ten hospitals have performed direct observation of compliance. Overall rate: 33,3%, (29,7% the first period; 35,9% for the second (p<0,05)). Alcohol-based products were used in 49,6% of occasions in which actions of HH occurred. The consumption ranged from 18,1 to 39,2 L/ 1000 patient-days (average 27,3 mL/1000 patient-days). Reminders were used and a local guide was performed and released.

The core team met in four occasions. Each facility was required to address a clear plan of activities for 5 may 2010. All centers have had registered to the 2009 WHO initiative “Save Lives: Clean your hands”.

## Conclusion

A clear improvement in HH practices was achieved. In order to get sustainability, new activities including clear commitment of leaders, patient involvement, and a system for ensuring HCW training are required.

## Disclosure of interest

None declared.

